# Supplementary material for: Knowledge-guided gene prioritization reveals new insights into the mechanisms of chemoresistance
Source: Genome Biol. 2017 Aug 11;18:153. doi: 10.1186/s13059-017-1282-3 (PMC5554409; doi:10.1186/s13059-017-1282-3)
Supplement: Supplementary file 1 — Supplemental methods and supplemental figures. (DOCX 22058 kb) [file 13059_2017_1282_MOESM1_ESM.docx]

**Supplemental Material for: Knowledge-guided gene prioritization reveals new insights into the mechanisms of chemoresistance**

**SUPPLEMENTAL METHODS**

**Data collection**

We obtained basal gene expression and drug response data on 284 lymphoblastoid cell lines (LCLs) from [1, 2]. The raw expression data consisting of 54,613 Affymetrix U133 Plus 2.0 Gene-ChIP probes were transformed using ${log}_{2}$ GC Robust Multi-array Averaging (GC-RMA) and mapped to ENSEMBL IDs of 17,303 genes. The drug response data consisted of half maximal effective concentration (EC50) of 24 cytotoxic treatments. This dataset is referred as the ‘LCL dataset’ throughout the manuscript.

We also obtained gene expression and drug response data on 624 cancer cell lines from 13 different tissue origins from the Genomics of Drug Sensitivity in Cancer (GDSC) database (release-5.0) [3]. The basal gene expression data consisting of RMA-normalized expression of 13,322 genes, obtained using Affymetrix U133A platform, were ${log}_{2}$ transformed making it consistent with the LCL dataset. The drug response data consisted of half maximal inhibitory concentration (IC50) of 139 cytotoxic drugs. This dataset is referred as the ‘GDSC dataset’ throughout the manuscript.

The main gene interaction network used in this manuscript was obtained form the STRING database [4]. This network consisted of genetic interactions, protein associations and protein colocalizations obtained experimentally. We did not use any computationally predicted interactions or interactions based on text mining to form this network. The final network consisted of approximately 1.48 M undirected weighted edges (relationships) among 15,589 nodes (genes).

**Convergence criterion for the RWR algorithm**

Let $T(\varepsilon)$ denote the number of iterations required to guarantee $\sum_{i=1}^{N_{n}} \boldsymbol{|v}^{\boldsymbol{(}t)}[i]\boldsymbol{-}\boldsymbol{v}^{\boldsymbol{(}t-1)}[i]|<\varepsilon$, where $\boldsymbol{v}^{\boldsymbol{(}t)}$ is the vector of equilibrium probabilities at iteration $t$, and $\boldsymbol{v}^{\boldsymbol{(}t)}[i]$ is its $i$th entry. We used $\text{min}(100, T(\varepsilon))$ as the convergence criterion, where$\varepsilon=$10E-8. In all cases, $T\left( \varepsilon\right)<100$ iterations was sufficient.

**Borda method with geometric mean used to aggregate ranked lists of genes**

Given $N_{r}$ ranked lists of$n$ genes, for each gene we calculated a set of $N_{r}$ Borda scores equal to $(n-r_{i}+1)$, where $r_{i}$ is the rank of the gene in the $i$th list. Then, we used the geometric mean of these $N_{r}$ Borda scores as the final ranking criterion.

**Set of parameters used to train the SVR in the cross validation scheme for prediction of drug response**

We used the support vector regression algorithm with Gaussian kernel from the scikit-learn library for python [5]. Hyperparameters of the SVR were learnt using a 4-fold cross validation applied inside the training set. We used two choices {0.1, 0.01} for ‘epsilon’ and seven choices {5E-6, 1.08E-5, 2.32E-5, 5E-5, 1.08E-4, 2.32E-4, 5E-4} for ‘gamma’.

**Parameters used to rank genes using Elastic Net**

We used the Elastic Net (EN) implementation from the scikit-learn library for python [5] (see http://scikit-learn.org/stable/modules/generated/sklearn.linear_model.ElasticNet.html). This library implements EN by minimizing the objective function,

$$\frac{1}{2n}\left\| \left. y-Xw \right\| \right._{2}^{2}+\alpha\rho\left\| w \right\|_{1}+\frac{1}{2}\alpha\left( 1-\rho\right)\left\| \left. w \right\| \right._{2}^{2},$$

where $n$ is the number of samples, $y$ is the response vector, $X$ is the feature matrix, $w$ is the unknown vector of coefficients, and $\alpha$ and $\rho$ are hyperparameters. To choose the hyperparameters, we used a 4-fold inner cross-validation (CV) (for each training set), and the best choices of hyperparameters were chosen to train a model and predict the response of the test set.

We considered two options for the set of hyperparameters from which the inner CV can choose:

Option 1: two choices for $\alpha$, {0.01, 0.05}, and four choices for $\rho$, {0.05, 0.1, 0.5, 1}.

Option 2: three choices for$\alpha$, {0.5, 1, 2}, and four choices for $\rho$, {0.05, 0.1, 0.5, 1}.

Note that $\rho=1$ results in LASSO objective function. The second option, in many cases, resulted in very few selected features and its performance was significantly worse than the first option (p-value = 4.6E-2 on the LCL dataset, one-sided Wilcoxon signed rank test on average SPCI for each drug). As a result, we did not use this option and only used option 1 for the analysis reported in the manuscript. We also tried a third option, in which both large and small values of $\alpha$ were considered, but the performance of this option was very similar to option 2 and worse than the first option.

In addition to the aforementioned choices, we also used the glment R-package [6] (the installation guide is available at https://web.stanford.edu/~hastie/glmnet/glmnet_alpha.html). This package uses the same objective function above and allows automatically choosing the best value for $\alpha$ using coordinate descent. We used $\rho=0.5$ to balance the strength of the two regularization terms and used the default values of other parameters in the “cv.glmnet” function. Using this setup, ProGENI-SVR outperformed EN-glmnet for the LCL dataset (p-value = 8.12 E-5, one-sided Wilcoxon signed rank test on the average SPCI for each drug). Similarly, using the GDSC dataset, ProGENI-SVR significantly outperformed EN-glmnet (p-value = 1.04 E-9, one-sided Wilcoxon signed rank test on the average SPCI for each drug).

**Bayesian multitask MKL:**

This algorithm is the winner of the DREAM 7 challenge [7] and uses multitask multiview learning to predict drug response. We downloaded the implementation of this code in R from https://github.com/mehmetgonen/bmtmkl. In our analysis, we only used the gene expression data for this algorithm and used a Gaussian kernel as described in [7]. We did not use other types of data that (such as methylation, copy number variation, etc.), since all other methods we considered in this manuscript only used gene expression data and also since it has been shown that gene expression data is the most informative type of data in drug response prediction using Bayesian multitask MKL [7]. However, one should note that the performance of all methods considered in this manuscript may improve upon utilizing other types of information.

**The escalation of concentrations used for each drug in cytotoxicity assays**

Cytotoxicity assays with the tumor cell lines were performed with the CellTiter 96® AQueous Non-Radioactive Cell Proliferation Assay (Promega Corporation, Madison, WI). Specifically, 90 μL of cells (5 × 103 cells) were plated into 96-well plates and were treated with increasing dose of specific drug. The escalation of concentrations for each drug is list below:

Doxorubicin: 0, 0.0156, 0.03125, 0.0625, 0.125, 0.25, 0.55, 1, 2, and 4 µmol/L;

Docetaxel: 0, 0.01, 0.1, 1, 10, 50, 100, 1000, 5000, and 10000 nmol/L;

Cisplatin: 0, 1.25, 2.5, 5, 10, 20, 40, 60, 80, and 100 µmol/L;

**Scaled probabilistic concordance index**

In order to evaluate the performance of different algorithms in predicting the drug response, we used the scaled probabilistic concordance index [7]. This measure is a modification of the concordance index (c-index), and has been developed specifically to evaluate the performance of drug response prediction algorithms, and has been used to score different teams in the DREAM 7 challenge. The c-index is a measure that can be used to compare the concordance between two ranked lists [8]. Given two ranked lists of items, this measure is calculated as the number of times the relative ranking of a pair of items match in both lists (ties are counted as 0.5) divided by the total number of pairs. One major drawback of this measure in evaluating drug response predictions is that the c-index does not take into account the variance within the values of the drug response. As a result, correctly predicting the relative standing of two cell lines with very similar drug response values has the same importance as correctly predicting the relative standing of two cell lines with very different drug response values.

To overcome this shortcoming, the probabilistic concordance index has been proposed [7]. Let $T = \{t_{1}, t_{2}{,\ldots,t}_{n}\}$ be the list of true response of $n$ cell-lines to a drug and let $P = \{p_{1}, p_{2}{,\ldots,p}_{n}\}$ be the predicted values for the drug response. In this representation, $t_{i}$ is the true response of cell line $i$ to the drug and $p_{i}$ is the predicted response of cell line $i$ to the drug. The pc-index (PCI) for these two lists is calculated as

$$PCI(T,P) = \frac{2}{n(n-1)}\sum_{i<j} f\left( t_{i}, t_{j},p_{i},p_{j}, \sigma_{d} \right)$$

where

$$f\left( t_{i}, t_{j},p_{i},p_{j}, \sigma_{d} \right) = \left\{ \begin{matrix} \frac{1}{2}\left( 1+\text{erf}\left( \frac{t_{i}-t_{j}}{2\sigma_{d}} \right) \right), & \text{if }\left( p_{i}>p_{j} \right) \\ 0.5 & \text{if }\left( p_{i}=p_{j} \right) \\ \frac{1}{2}\left( 1+\text{erf}\left( \frac{t_{j}-t_{i}}{2\sigma_{d}} \right) \right), & \text{if }\left( p_{i}<p_{j} \right) \end{matrix} \right.$$

and

$$\text{erf}\left( x \right)=\frac{2}{\sqrt{\pi}}\int_{0}^{x} e^{-y^{2}}dy$$

is the error function and $\sigma_{d}$ is the standard deviation of $T$.

In our evaluations, we used the scaled version of the pc-index (SPCI), which is calculated for each drug as $SPCI = \frac{PCI-{PCI}_{min}}{{PCI}_{max}-{PCI}_{min}}$. This scaling ensures that the SPCI for different drugs are comparable. Note that SPCI is always between 0 and 1.

**Forming dense biclusters of genes and drugs**

To obtain the biclusters, we first formed a binary drugs x genes matrix indicating the top 500 genes identified for each drug. Then, columns (genes) with variance larger than 0.1 were kept (total of 1177 genes). This filtering step was performed to keep the most informative genes (i.e. genes that can differentiate groups of drugs from each other). Next, agglomerative clustering using the Ward algorithm was performed on columns and rows independently. To perform pathway enrichment analysis we identified four dense biclusters using the following procedure. Since the drugs x genes matrix is a binary matrix with a value of 1 in the (i,j)th cell representing the presence of gene j among the top ranked genes for drug i, we focused only on dense biclusters. The agglomerative clustering on the genes (columns) identified three major clusters (represented by colors red, green and blue in Fig. 5A). Biclusters B and C in Fig. 5 were chosen since they are the densest biclusters representing two of the main gene clusters (out of 3) and which also represent two of the main drug clusters (out of 3) (represented by colors green and red in the dendogram both on rows and columns). However, for the third gene cluster, represented by color blue in the dendogram (top right corner of the Fig. 5A), there was no single representative dense bicluster. Therefore, we continued further down the dendogram sub-tree until we obtained 4 smaller gene clusters. Two of these gene clusters had dense representative biclusters, which we chose as clusters D and E. So to summarize, the procedure for choosing bicluster B, C, D, E was to go down the dendogram corresponding to the gene clusters one level at a time. If there exists a dense bicluster for that gene cluster, include that in our set; if not, continue the process until such dense biclusters are found.

**Test of normality for using t-test in siRNA knockdown results**

The normality assumption for t-tests used in Fig. 4 was evaluated using the Cramer-von Mises test [9] (see Additional file 4 for results of this test). Out of the 38 cases, 2 p-values were smaller than 0.05 (i.e. the normality assumption was rejected), however since under the null hypothesis that all of the 38 cases are distributed according to a Normal distribution, with α = 0.05 in average we expect to see approximately 2 p-values smaller than alpha, we believe that t-test can be applied in those two scenarios as well.

**SUPPLEMENTAL FIGURES:**

Figure S1: The performance of drug sensitivity prediction based on ProGENI-SVR (y-axis) compared to the PCC-SVR scheme (x-axis) using the LCL dataset. Each point in the scatter plot corresponds to one random choice of training/test set. The color of each point represents the density of points in that region: a dark red color on a point means that the point is surrounded by many other points, while a blue color on a point means that the point is isolated. The FDR is calculated using a two-sided Wilcoxon signed rank test adjusted for multiple tests.

Figure S2: The performance of drug sensitivity prediction based on ProGENI-SVR (y-axis) compared to the EN-SVR scheme (x-axis) using the LCL dataset. Each point in the scatter plot corresponds to one random choice of training/test set. The color of each point represents the density of points in that region: a dark red color on a point means that the point is surrounded by many other points, while a blue color on a point means that the point is isolated. The FDR is calculated using a two-sided Wilcoxon signed rank test adjusted for multiple tests.

Figure S3: The performance of drug sensitivity prediction based on ProGENI-SVR (y-axis) compared to the EN scheme (x-axis) using the LCL dataset. Each point in the scatter plot corresponds to one random choice of training/test set. The color of each point represents the density of points in that region: a dark red color on a point means that the point is surrounded by many other points, while a blue color on a point means that the point is isolated. The FDR is calculated using a two-sided Wilcoxon signed rank test adjusted for multiple tests.

Figure S4: The performance of drug sensitivity prediction based on ProGENI-SVR (y-axis) compared to the Bayesian Multitask-MKL scheme (x-axis) using the LCL dataset. Each point in the scatter plot corresponds to one random choice of training/test set. The color of each point represents the density of points in that region: a dark red color on a point means that the point is surrounded by many other points, while a blue color on a point means that the point is isolated. The FDR is calculated using a two-sided Wilcoxon signed rank test adjusted for multiple tests.

Figure S5: The performance of drug sensitivity prediction based on ProGENI-SVR using the BDI network (y-axis) compared to the PCC-SVR scheme (x-axis) using the LCL dataset. Each point in the scatter plot corresponds to one random choice of training/test set. The color of each point represents the density of points in that region: a dark red color on a point means that the point is surrounded by many other points, while a blue color on a point means that the point is isolated. The FDR is calculated using a two-sided Wilcoxon signed rank test adjusted for multiple tests.

Figure S6: The performance of drug sensitivity prediction based on ProGENI-SVR using the STRING network (y-axis) compared to the ProGENI-SVR using the BDI network (x-axis) using the LCL dataset. Each point in the scatter plot corresponds to one random choice of training/test set. The color of each point represents the density of points in that region: a dark red color on a point means that the point is surrounded by many other points, while a blue color on a point means that the point is isolated. The FDR is calculated using a two-sided Wilcoxon signed rank test adjusted for multiple tests.

Figure S7: The performance of drug sensitivity prediction based on ProGENI-SVR using the subnetwork of BDI corresponding to molecular associations (y-axis) compared to the PCC-SVR scheme (x-axis) using the LCL dataset. Each point in the scatter plot corresponds to one random choice of training/test set. The color of each point represents the density of points in that region: a dark red color on a point means that the point is surrounded by many other points, while a blue color on a point means that the point is isolated. The FDR is calculated using a two-sided Wilcoxon signed rank test adjusted for multiple tests.

Figure S8: The performance of drug sensitivity prediction based on ProGENI-SVR using the subnetwork of BDI corresponding to colocalization (y-axis) compared to the PCC-SVR scheme (x-axis) using the LCL dataset. Each point in the scatter plot corresponds to one random choice of training/test set. The color of each point represents the density of points in that region: a dark red color on a point means that the point is surrounded by many other points, while a blue color on a point means that the point is isolated. The FDR is calculated using a two-sided Wilcoxon signed rank test adjusted for multiple tests.

Figure S9: The performance of drug sensitivity prediction based on ProGENI-SVR using the subnetwork of BDI corresponding to genetic interactions (y-axis) compared to the PCC-SVR scheme (x-axis) using the LCL dataset. Each point in the scatter plot corresponds to one random choice of training/test set. The color of each point represents the density of points in that region: a dark red color on a point means that the point is surrounded by many other points, while a blue color on a point means that the point is isolated. The FDR is calculated using a two-sided Wilcoxon signed rank test adjusted for multiple tests.

Figure S10: Dosage-response curves for the genes identified using Robust-ProGENI which did not show significant change compared to control for a) cisplatin, b) docetaxel, and c) doxorobicin in BT549 cell lines using a two-tailed unpaired t-test.

Figure S11: Dosage-response curves for the genes identified using Robust-ProGENI which did not show significant change compared to control for a) cisplatin, b) docetaxel, and c) doxorobicin in MDA-MB-231 cell lines using a two-tailed unpaired t-test.

**References**

1. Hanson C, Cairns J, Wang L, Sinha S. Computational discovery of transcription factors associated with drug response. Pharmacogenomics J 2015.

2. Niu N, Qin Y, Fridley BL, Hou J, Kalari KR, Zhu M, Wu TY, Jenkins GD, Batzler A, Wang L. Radiation pharmacogenomics: a genome-wide association approach to identify radiation response biomarkers using human lymphoblastoid cell lines. Genome Res 2010;20:1482-1492.

3. Yang W, Soares J, Greninger P, Edelman EJ, Lightfoot H, Forbes S, Bindal N, Beare D, Smith JA, Thompson IR, et al. Genomics of Drug Sensitivity in Cancer (GDSC): a resource for therapeutic biomarker discovery in cancer cells. Nucleic Acids Res 2013;41:D955-961.

4. Szklarczyk D, Franceschini A, Wyder S, Forslund K, Heller D, Huerta-Cepas J, Simonovic M, Roth A, Santos A, Tsafou KP, et al. STRING v10: protein-protein interaction networks, integrated over the tree of life. Nucleic Acids Res 2015;43:D447-452.

5. Pedregosa F, Varoquaux G, Gramfort A, Michel V, Thirion B, Grisel O, Blondel M, Prettenhofer P, Weiss R, Dubourg V. Scikit-learn: Machine learning in Python. Journal of Machine Learning Research 2011;12:2825-2830.

6. Friedman J, Hastie T, Tibshirani R. Regularization Paths for Generalized Linear Models via Coordinate Descent. J Stat Softw 2010;33:1-22.

7. Costello JC, Heiser LM, Georgii E, Gönen M, Menden MP, Wang NJ, Bansal M, Hintsanen P, Khan SA, Mpindi J-P. A community effort to assess and improve drug sensitivity prediction algorithms. Nature biotechnology 2014;32:1202-1212.

8. Harrell F: *Regression modeling strategies: with applications to linear models, logistic and ordinal regression, and survival analysis.* Springer; 2015.

9. Stephens MA. Tests based on EDF statistics. Goodness-of-fit Techniques 1986;68:97-193.
